# Tumor suppressor role of genes involved in circadian clock control

**DOI:** 10.17179/excli2019-2072

**Published:** 2019-12-19

**Authors:** Reham Hassan

**Affiliations:** 1 Forensic Medicine and Toxicology Department, Faculty of Veterinary Medicine, South Valley University, Qena, Egypt

## ⁯⁯⁯⁯⁯

In recent years evidence has accumulated that genes involved in circadian clock control play a role as tumor suppressors. Strong evidence has been presented by Broadberry and colleagues who published an article about disrupted circadian clocks in breast cancer (Broadberry et al., 2018[1]). The authors studied primary mammary epithelial cells from normal breast tissue and epithelial cells from breast carcinomas of the same patients. They transduced the cells with the luciferase clock reporter PER2:Luc, which is known to show a robust ~24 h rhythm in normal epithelial cells (Yang et al., 2017[20]). The normal epithelial breast cells showed the expected cycling. However, epithelial cells from the cancer tissue of the same individuals showed a disrupted rhythm with a much lower amplitude (Broadberry et al., 2018[1]). This study confirms previous studies presenting evidence for a tumor suppressor role of the circadian clock (Gery and Koeffler, 2010[7]; Grundy et al., 2013[9]; Fu and Lee, 2003[6]; Mormont and Lévi, 1997[15]; Filipski et al., 2002[5]). Loss of clock genes has been shown to be associated with worse prognosis in breast cancer (Cadenas et al., 2014[3]). Coordinated co-expression of clock genes (e.g. PER2-PER3 and CRY2-PER3) is maintained in estrogen receptor positive and HER2 negative carcinomas but is compromised in more aggressive tumors (Cadenas et al., 2014[3]). Genes relevant for progression of breast carcinomas have been shown to be associated with proliferation (Siggelkow et al., 2012[18]), the cellular and humoral immune system (Schmidt et al., 2008[16], 2012[17]; Heimes et al., 2017[10][11]; Lohr et al., 2013[13]; Godoy et al., 2014[8]), anti-oxidative and anti-apoptotic factors (Hellwig et al., 2016[12]; Cadenas et al., 2010[2]) and altered metabolism (Cadenas et al., 2019[4]; Marchan et al., 2017[14]; Stewart et al., 2012[19]). Although the loss of circadian clock gene expression and its association with tumor prognosis has clearly been shown, the mechanisms of their tumor suppressive effect still need to be elucidated. 
